# Circumcision

**Published:** 1900-07-28

**Authors:** 


### CIRCUMCISION.

Whatever may have been the origin of the ancient rite of circumcision, it can hardly be open to question that, if it is to continue, such interference with the human frame should be performed with all the safeguards which modern surgical science affords for the protection of its little victims. Whatever the religious ceremonies by which it is surrounded, the actual surgical part of the performance should be done by people who possess some surgical qualification. A writer in the *Polyclinic* describes the process as it is now performed by the Jew operators. In common with other nations which practise this rite, they remove the skin only, and do not cut the mucous membrane. Above all they never touch the frenum, and to this, no doubt, is due the absence of bleeding, an event in regard to which they seem to have no anxiety. Their method is to draw the skin well forward into the grip of blunt blades, and then to shave it off, leaving the glans still covered by a hood of mucous membrane. The latter is then torn up along the dorsum by the thumb-nails, and is turned over on each side and left. Thus the frenum is untouched, and the incised wound is in skin alone. The only precaution taken by the Jewish operators which is in the least an antiseptic one is that the operator having first taken wine into his mouth subsequently sucks the infant's penis. The organ is wrapped in lint, dry or oiled, or it may be left uncovered. Possibly it might be well if a mutual concession were made in regard to the performance of circumcision. So far as the arrangement of the incision is concerned we might well take a leaf out of the Jewish book, and, by avoiding the frenum, escape what is undoubtedly a definite risk in the operation as it is commonly performed by surgeons, namely, that of hæmorrhage. On the other hand, it certainly would be well that the little operation should have those aseptic surroundings which are now regarded as essential in all surgical proceedings. There can be little doubt that, if all risk could be eliminated, a much wider extension of the practice of circumcision, among people who would accept it for sanitary reasons rather than as a religious rite, would be attended with many benefits, and would be preventive of much disease and suffering.

